# The role of microfinance institutions on women’s entrepreneurship development

**DOI:** 10.1186/s13731-023-00285-0

**Published:** 2023-03-20

**Authors:** Abraham Abebe, Meketaw Kegne

**Affiliations:** 1 Department of Management, School of Graduate Studies, Assosa University, P. BOX 18, Assosa, Ethiopia; 2 Department of Management, College of Business and Economics, Assosa University, Assosa, Ethiopia

**Keywords:** Women entrepreneurship, Microfinance institution, Access to finance, Saving, Business support, Skill development

## Abstract

This study investigates the role of microfinance services on women's entrepreneurship development in Assosa town. The study employed both descriptive and explanatory designs and a quantitative research approach. The study targeted 352 women clients of Assosa Woreda Microfinance Institution, and 165 samples were selected using a simple random sampling technique. The data were collected through a questionnaire and analyzed through the statistical package for social science (SPSS) 26 software. The findings from the descriptive mean analysis indicate that the microfinance institution financial and non-financial services offered were found unable to significantly empower disadvantaged and poor women by improving their livelihood and development of their business. The correlation result also indicated a positive and significant association between saving practice, access to credit, skill development training, and the development of women entrepreneurs. Finally, the regression result saving and the credit or loan services of the microfinance institution service have the most decisive influence on women's entrepreneurship development.

## Introduction

Microfinance is a no-collateral loan given to the poorest people in rural areas where traditional banks have disregarded these communities due to a lack of collateral and regulatory systems that make borrowers' loan repayments exceedingly difficult, if not impossible (Minai et al., 2021). Microfinance services also benefit women and play an essential role in their empowerment. Economic empowerment is designed to generate increased self-esteem, respect, and other forms of empowerment for women beneficiaries. More specifically, microfinance services as development organizations are to service the financial needs of un-served or underserved markets as a means of meeting development. It includes reducing poverty, allowing them to earn an independent income, empowering women or other disadvantaged population groups to create employment (Ledgerwood, 2000; Tariq & Sangmi, 2019).

Entrepreneurship is an effective method for creating job opportunities in any given country, and female entrepreneurs play an essential role in any given country (Nawaz, 2018). Furthermore, Ward (2009) defines entrepreneurship as an individual's ability and action to start, establish, and manage a business enterprise despite any obstacles to profit. Entrepreneurship, therefore, involves the planning, initiating, and managing a new business that offers a product or service (Karlan & Valdivia, 2011). In this regard, an entrepreneur, therefore, can be defined as a person who organizes and runs an enterprise, especially a business, usually with considerable ingenuity and risk rather than working as an employee (Jayawarna et al., 2013). According to the International Labor Organization (ILO, 2007), the only disadvantage of the entrepreneurial culture is that the role of women in entrepreneurship has not been recognized; the majority of entrepreneurs are men, with a small number of women. Women's entrepreneurial skills have not been fully utilized, or such skills have not been recognized and utilized. As a result, women predominantly suffer from poverty in many developing countries, and gender inequalities in developing societies inhibit economic growth and national development (Elson, 2009). Currently, more than ever, it is acknowledged by many, including those in government, in the development community, and civil society, that all-inclusive development, sustainable wealth creation, and employment can only be achievable if women are enabled to participate in the small business sector. Studies have shown that women can stimulate economic growth and thereby create employment when they are assisted in generating income and accruing assets. Furthermore, women are more environmentally conscious, aware of resource efficiency, are more likely to reuse, and make long-term decisions for their families and businesses (Ahl & Marlow, 2012).

Therefore, putting resources in the hands of poor women while promoting gender equality in the household and society results in significant development payoffs. However, as most aspiring businesswomen cannot access credit from the established banking system or have their savings to rely on, they resort to microfinance services (MFI) for credit (Kuzilwa, 2005). Therefore, the primary goal of microfinance is to provide small-scale loans to women in rural communities and educate them on how to succeed in business or other fields of endeavor (Vossenberg, 2023). Further, microfinance services are attractive to most women entrepreneurs as they do not have stringent conditions in the banking sector. In most cases, they encourage entrepreneurs to save either individually or as members of a group, which the microfinance institution uses to lend back to the clients (Mkpado & Arene, 2007).

Microfinance was introduced in Ethiopia, with the proclamation in 1996 serving as the catalyst. Before the proclamation, it also stated that only a few non-governmental organizations (NGOs) and the Development Bank of Ethiopia provided limited and isolated microfinance services ad hoc. However, this is not regarded as well-organized or capable of running continuously and sustainably (Amha, 2012). The heavily subsidized nature of many NGO programmers, with low-interest rates for credit, high default rates, and a lack of saving products, was a significant source of criticism. In response, the government enacted legislation governing the licensing and supervision of banking businesses (Proclamation No. 84/1994), allowing private financial institutions and breaking the state monopoly in the banking sector (Gobezie, 2005). Shortly after, the first microfinance law (Proclamation No. 40/1996) was enacted to protect small depositors, ensure stability, and promote MFI efficiency (Fite, 2013). Furthermore, it integrated all MFIs into the monetary and financial system, allowing deposit-taking while delegating regulation and supervision to the National Bank of Ethiopia (NBE).

Even though, as a result of microfinance programs, millions of women worldwide have been brought into commercial, economic activities that they could previously not have participated in. However, fewer women than men manage and run businesses in many parts of the world. Many women-run businesses are in less profitable areas or grow more slowly, and in most cases, they are likely to fail. According to the International Fund for Agricultural Development (IFAD), women involved in microfinance, either as borrowers or savers, gain a level of autonomy that enables them to make independent decisions and raise their status. However, despite this fact, and although businesswomen have in the past few years been joining MFIs with the main aim of economically empowering themselves, very little progress has been made (Rogaly, 2009). Generally, women have made progress in attracting venture capital (Brush et al., 2018). However, despite the economic importance and the number of women in self-employment is increasing throughout the world, their number still lags behind that of male entrepreneurs (Dabic et al., 2012; Verheul et al., 2006). Therefore, recognizing the importance of women's economic empowerment, this study investigated the role of microfinance services on women's entrepreneurship development in Assosa town credit and saving institutions, one of the earliest micro-financing institutions in Assosa Woreda.

## Literature review and hypothesis development

### Microfinance and women entrepreneurship

Microfinance is a term used to refer to the provision of financial services to clients who are not benefited from the traditional banking system because of their lower economic status. The financial services will most commonly take the form of loans and savings by removing collateral requirements and creating a banking system based on mutual trust. According to this simple definition, microfinance targets low-income people who have no access to the formal lending system (Rehman et al., 2015). It is generally dedicated to needy communities to support economic development by expanding their entrepreneurial activities. Capacity building services, management, vocational skills training, consultancy, advisory services, marketing assistance, information, technological development, transfer, and business linkage promotion are examples of the latter (Bruton et al., 2011; Khavul et al*.*, 2013).

The growing number of female entrepreneurs in developing countries has piqued the interest of academics and the related industry. Donors, international public institutions, governmental authorities, non-governmental organizations (NGOs), private corporations, charities, research institutes, and businesses have launched programs or policies to encourage and support female entrepreneurs. They initiate programs to improve entrepreneurial skill capacity, strengthen women's networks, facilitate funding and training, or create policies to encourage better startups and business expansion. They are unanimous in their belief that women's entrepreneurship is critical to growth and development. Women are less likely than men to be involved in entrepreneurial activity globally (Vossenberg, 2023).

Microcredit is about much more than simply having access to money. It is about women gaining control over their means of subsistence. It is about women rising above poverty and vulnerability. It is about women gaining economic and political power in their homes, villages, and countries. As a result, to promote women's entrepreneurship development, microfinance must assist poor women in meeting both their daily needs and their strategic gender interests. It is recognized that strategic gender interests are at the heart of patriarchal power structures: the abolition of a coercive gender division of labor; unequal control over resources; the abolition of male violence, women's control over their bodies, the establishment of political equality, and the abolition of sexual exploitation.

Women Entrepreneurs means the women or a group of women who initiate, organize and operate a business enterprise. Therefore, a woman entrepreneur's business growth is a significant issue in entrepreneurship. Despite its significance, not much work has been done to study the growth of women-owned enterprises until recently. There was a lack of cumulative knowledge to adequately conceptualize and build explanatory theories on women-owned enterprises' growth process (Brush & Cooper, 2012). Most of the work conducted was on women's motivation to start a business and the subsequent effect of those motivations on growth performance and the effect of size and sector on business development (Du Rietz & Henrekson, 2000). The main objective of microfinance services is to allow people to access financial services to engage in income-generating activities. Though women have a crucial role in their communities and families' economic development, hurdles such as poverty, joblessness, low earnings, and societal bias have obstructed them from effectively performing that role. It is now clear that women entrepreneurs cannot easily access finance to facilitate their entrepreneurial activities in some countries, unlike their male counterparts.

### Gender-based microfinance delivery

Women's access to financial resources has been substantially increasing over the years. However, their ability to benefit from finance is influenced by gender-related disadvantages (Skarlatos, 2004). In addition, despite their growing capacities, some microfinance services provide a decreasing percentage of loans to women. The loan size provided to women also appears to be smaller than men, although both participate in the same program and belong to the same community. In addition to women's poverty levels, social discrimination against women results in smaller loan sizes than men (Cheston & Kuhn, 2002).

Furthermore, there are only a limited number of women in the leadership of microfinance institutions, which might be one reason for the limited loan access. However, regardless of the odds, microfinance programs still can transform power relations and empower the poor. Although microfinance does not address all the impediments to women's empowerment, it can contribute to their empowerment if adequately implemented. Hence, creating gender-based policy involves a process through which an institution re-examines all of the underlying structures and assumptions about gender roles, rights, and responsibilities that have historically discriminated against women as borrowers and employees. It is also essential for microfinance services to set guidelines on employee recruitment, promotion, roles, and responsibilities. In this regard, the formulation and enforcement of the guidelines are expected to bring about positive social changes. New and strengthening existing microcredit mechanisms and microfinance institutions must be undertaken to enhance credit outreach (Cheston & Kuhn, 2002). In addition, other supportive measures should be undertaken to ensure an adequate flow of funds. They promote women's political participation in government and national and local party politics and support women's involvement in NGOs and women's movements. Generally, although women are found in large numbers in lower-level positions in public administration political parties, trade unions, and business, their representation in chief executive and economic areas is poor.

### Empirical literature

#### Challenges of women entrepreneurs

Amine and Staub (2009) demonstrate that women entrepreneurs in sub-Saharan Africa face several challenges due to socio-cultural, economic, legal, political, and technological environments. Also, unfavorable conditions in local regulatory, normative, and cognitive systems place additional burdens on them. Akanmu et al. (2018), inadequate finance, a lack of access to raw materials, maladministration, insufficient market information, and a lack of good coordination are the main challenges that partners face in developing entrepreneurship among members. According to Ayatakshi-Endow and Steele (2021), social relationships are the most important factors influencing women's entrepreneurial experiences in the current COVID-19 crisis. They also stated that women entrepreneurs seek support in their social relationships, both within and outside the family, even though gender roles are acknowledged as a significant challenge. According to a study conducted by Bonin et al. (2021), in India to assess the impact of COVID-19, women entrepreneurs did complain that accessing funds was already difficult before the crisis due to gender bias. These results support that the main barriers to female entrepreneurship in India are gender discrimination. According to Ferdousi (2015), the leading financial and management-related barriers to women for starting microenterprises are lack of capital, entrepreneurial, and management skills, respectively.

Further, (Shkodra et al., 2021) found that finances continue to be their most challenging despite the importance of developing women's businesses. Mauchi et al. (2014) conclude that conflicts in the work–life interface, poor networking, educational and managerial skills, adequate training, low financing and capital-base, raw materials accessing, and costs, are the key challenges facing female entrepreneurs. Salum (2014) found that MFIs play a significant role in enhancing micro-entrepreneurs in Tanzania. MFIs provide loan services, consultation, training, and business monitoring services. Also, the majority of the respondents (51.67%) identified that a high-interest rate is a strong barrier in impeding entrepreneur development. Also, the study found that the majority of the respondents (60%) identified high-interest rates as major constraints facing micro-entrepreneurs. Moreover, the study found that the majority of the respondents (58.3%) revealed that poor management is a strong challenge for microfinance services in providing loans to micro-entrepreneurs.

#### Access to credit and growth of women entrepreneurial ventures

The availability of capital is essential for entrepreneurship as it lays the foundation for the business (Verheul et al., 2006), and most scholars have concluded that microfinance schemes embarked in many developing countries have yielded positive results (Baruah & Bezbaruah, 2020; Elson, 2009; Mauchi et al., 2014; Rehman et al., 2015). However, accessing financial resources is a task that many entrepreneurs face. It is argued that in both the developed and developing world, women entrepreneurs face additional challenges in accessing finance (Khandker and Samad, 2013). Mansor and Mat (2010) studied 436 women business establishments in the state of Terengganu in Malaysia found that women were observed to be constrained in their access to formal bank credit as they are perceived to be risky borrowers due to a lack of adequate collateral. This perspective is more pronounced in cultural settings where the women have less land and property rights than men and cannot offer the banks the preferred type of collateral, usually land and property. This difficulty encountered by women in obtaining financial resources induces them to choose low capital-intensive activities, like those in the services sector (Bruni et al., 2004). Women were also more likely to mention financial constraints, which likely reflected their poor financial performance in the previous fiscal year, as well as possibly greater under-capitalization at startup and a lower ability to raise external funds (Aidis et al., 2007)**.** Therefore, the microcredit service is significantly related to women-owned businesses, and the following hypothesis was developed based on these premises.

##### H_1_

Access to credit positively related to women entrepreneurs' development.

#### Influence of savings on the growth of women entrepreneurial ventures

In most nations, microfinance institutions offer both financial and non-financial services. The financial service includes small business loans to low-income clients, savings, insurance, mortgages, and retirement plans for those denied such services by traditional banking and financial institutions. Hence, saving is an integral part of development since it is responsible for securing the income that can also be pumped back into the business or it can be used to secure a loan Mkpado and Arene (2007) stated the need for savings for the protection of income and at the same time to use for securing loans and re-investment in the business. A study by Minniti and Naude (2010) wanted to understand why many women-owned SMEs had failed to reduce reliance on external sources despite receiving financial and other assistance from various donors. He also argues that women-run enterprises would create fewer jobs and experience a higher failure rate or retardation than men-run ones due to management constraints. Salum (2014) studied the role of microfinance services on entrepreneurial development in Dare Salaam, Tanzania, with a sample of 120 respondents. The study found that MFIs play a significant role in enhancing micro-entrepreneurs. MFIs provide loan services, consultation, training, and business monitoring services. The study found that most of the respondents (51.67%) identified that a high-interest rate is a strong barrier in impeding entrepreneur development. Also, the study found that the majority of the respondents (60%) identified high-interest rates as major constraints facing micro-entrepreneurs.

Moreover, the study found that the majority of the respondents (58.3%) revealed that poor management is a strong challenge for microfinance services in providing loans to micro-entrepreneurs. Further, Mkpado and Arene (2007) indicated that savings as a microfinance factor enable people with few assets to save because they can make weekly savings and contribute to group savings. Microfinance institutions mobilize such savings for further lending to other clients. It should also be noted that the non-material benefits of saving for low-income micro-entrepreneurs include, among other things, the fact that saving promotes the borrowers' responsibility and self-help, as well as familiarizes them with timely repayment. Therefore, the saving service of a microfinance institution is significantly related to the development of women-owned businesses, and the following hypothesis was developed based on these premises.

##### H_2_

The saving service is positively related to women entrepreneurs' development.

#### Training programs and the growth of women entrepreneurial ventures

It is generally believed that organizing effective entrepreneurship education programs or implementing appropriate activities/projects in existing business schools to promote students' success in an entrepreneurial career is one way to increase participation in entrepreneurial activities (Dabic et al., 2012). Lack of education and training hinders these developments, greatly affecting women's performance. Considering the uncommon situations of most women in the third world countries who are stuck in poverty, low educational levels, and other societal discriminations, training is a critical element in the MFI where women can be provided with adequate training and be exposed to experiences required to run business (Porter & Nagarajan, 2005).

As a result of these problems, microfinance institutions, particularly in developing countries, provide non-financial services in addition to their primary role of providing financial services to bridge the skill and knowledge gaps of their clients. Salum (2014) indicated that in Tanzania, MFIs offer consultation, training, and business monitoring services in addition to loan services. The study undertaken in the UK indicates that there is an important and direct relationship between training and a firm s performance. Training increases with firm size, but there is also a relationship between training and growth performance in turnover. Also, their findings revealed that higher levels of education for women might increase their chances of receiving funding (Carter et al., 2003). Prior business-related training is also vital for women's entrepreneurship success in Nigeria (Isidore, 2011). The "size of the business sector" and "health and primary education" are critical factors in fostering entrepreneurship in developing countries (Casero et al., 2013). Furthermore, a lack of skills and knowledge in running a business is a major factor in women running businesses. It was also realized that there is a meaningful relationship between training, on the one hand, and the rate of income, assets, and savings of female entrepreneurs, on the other hand on the study conducted in Jordan (Thaher et al., 2021). Despite their significant role, women frequently lack specific technical skills, discouraging them from starting businesses in the manufacturing and high-tech sectors and reducing their chances of survival in those sectors (Bruni et al., 2004). Therefore, a microfinance institution's business skill and management knowledge training service is significantly related to developing women-owned businesses. The following hypothesis was developed based on these premises.

##### H_3_

The skill development training is positively related to women entrepreneurs' development.

#### Business support and growth of women entrepreneurial ventures

Women entrepreneurs with a diverse or large set of ties in their network may connect to different parts of a social system and open information channels inaccessible to those with a small set of direct network ties. Women business owners who diversify their networks gain social capital and increase their chances of obtaining equity financing (Carter et al., 2003). Hence, business support related to networking and market linkage has a significant benefit to the success of women business owners. Therefore, in addition to the microcredit, savings, and training services provided by MFIs, it is critical to provide business support in various ways to overcome experience-related barriers during startup. In a study conducted by Thaher et al. (2021), providing incentives, psychological support, creating marketing linkage, and periodic monitoring and evaluation are positively related to women entrepreneurs' success in Jordan.

Furthermore, women's entrepreneurship is associated with gendered roles, but it is aided by social relationships both within and outside the family. The two aspects of family and business are strongly intertwined (Ayatakshi-Endow & Steele, 2021). Women, in general, have less access to the more powerful male networks. Therefore, family members may be required to broaden the network by paving the way into the male network. Business associations may play a vital role in connecting women entrepreneurs with other entrepreneurs (Aidis et al., 2007). Generally, non-financial services that assist women micro-entrepreneurs are enterprise development services. Business training, market linkage and technology services, skill development programs are examples of these. Therefore, microfinance institutions should be encouraged to strengthen and expand this support service to their clients. Since it's significantly related to the survival and development of women-owned business enterprises, the following hypothesis was developed based on these premises.

##### H_4_

The business support program is positively related to women entrepreneurs' development.

#### Microfinance services and women entrepreneurship development

Women are active participants in the world economy, and it is critical to understand how they contribute and the opportunities and barriers that exist in such environments (Greene et al., 2001). Despite the important role that women entrepreneurs play in the economic development of their families and countries, it has been discovered that women entrepreneurs perform poorly in business when compared to their male counterparts (Scott Shane, 2004). Many studies have been conducted on Microfinance Institutions (MFIs) that impact the success and development of women entrepreneurs. In a study conducted by Aidis et al. (2007), in Ukraine and Lithuania, only a minority of women-owned businesses in either country appeared to generate a surplus, a significantly worse performance pattern than their male-owned counterparts. Moreover, In Lithuania, most female SME owners reported not making enough to cover their living expenses, suggesting that many are operating close to the margin of economic viability. Adams and Barthlomew (2010) conducted a descriptive to examine the effect of microfinance on maize farmers in Nkoranza, Ghana. The finding indicated that microfinance had a minimal role in clients Also, lack of credit, savings, education or training, and social capital are the main factors that normally influence entrepreneurial performance (Scott Shane, 2004). Littlefield et al. (2003), in a study conducted in Nepal, indicated that women entrepreneurs who have access to MFIs, are economically empowered, are confident, firm, participate in communal decision-making, and are in a position of fighting against gender inequities. They also found out that 68% of their respondents were now making decisions that their husbands previously made. Khan et al. (2018), on the empirical study conducted in Pakistan, discovered a significant improvement in the social and economic status of women entrepreneurs and concluded that the overall effect of rural microcredit was positive in empowering women entrepreneurs and fostering innovation in the study area.

Furthermore, the extent to which MFIs have influenced the development of women's businesses in Kosovo will be understood from the recent empirical research conducted with 200 women entrepreneurs in Kosovo (Shkodra et al., 2021). The finding shows that MFI loans positively impacted the performance of women's businesses in terms of fixed assets, income, and household expenses. On the other hand, in the study conducted in Nigeria, the cooperative microfinance service enabled women entrepreneurs to alleviate poverty, acquire various skills, and create jobs. Further, it significantly contributed to the development of their community, made women entrepreneurs self-sufficient, and a meaningful living out of life (Akanmu et al., 2018). Therefore, the microfinance institution service is extremely beneficial to the development of women who own businesses and based on these premises, the following hypothesis was developed.

##### H_5_

Microfinance institution service positively impacts women entrepreneurs' development.

### Theoretical framework of the study

**Fig. 1:**
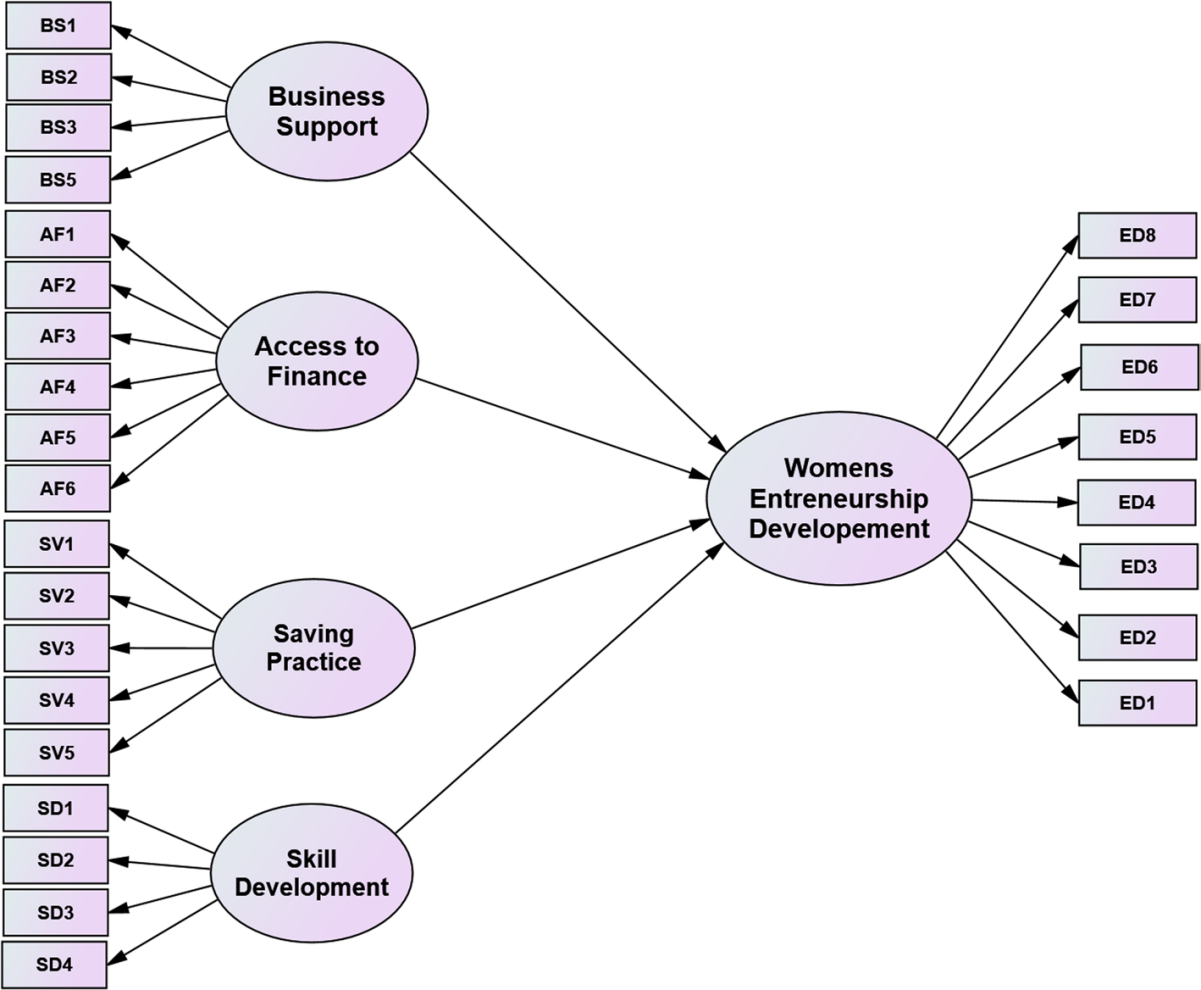
Theoretical framework of the study

The above literature review can be concluded that MFI's financial and non-financial service directly affects the development of women entrepreneurs. Therefore, provision of loan, saving, business skill development training, and business support services plays a significant role to women entrepreneurs in their daily lives, including economic independence and decision-making power in the household. Figure 1 depicts the theoretical framework developed from previous literatures.

## Research methods

### Research design and approach

The study employed both descriptive and explanatory research designs. This study described and assessed the practice of microfinance institution service and perceived values and importance on women's entrepreneurship development in Assosa town. Second, the study was also employed an explanatory research design to examine the causal relationship between microfinance services and women's entrepreneurship development in Assosa town. Moreover, the study was also utilized cross-sectional because all relevant data were collected at a single point in time. Further, a quantitative research approach addressed the study's main objectives. Creswell (2014), quantitative research provided a level of valid and repeatable results.

### Target population and participants of the study

The study targeted 352 women clients of Assosa Woreda Microfinance Institution. Therefore, 165 samples were included in the study using a simple random sampling technique.

### Sources of data and data collection method

The primary data were collected through a structured questionnaire. The questionnaire was directly distributed to 165 women clients of Assosa town Microfinance Institutions. However, with most of the respondents being barely able to read and write English and obtain accurate data, the final version of the survey questionnaire was also translated from English into the local language called Amharic. Further, the secondary data related to women clients micro and small enterprises and other data were collected from the microfinance institution's annual reports and bulletins.

### Reliability of the instrument

To determine the internal consistency of the measurement, a pilot study was carried out. The pilot test was executed with a sample size of 37 respondents and tested for reliability. Reliability analysis was subsequently done using Cronbach's Alpha and composite reliability, which measures the internal consistency that particular item within a scale measures the same variable. Cronbach's alpha was calculated by applying SPSS 26 to check the reliability of the measurement instrument. Reliability coefficients alpha range from 0.00 to 1.00, where the values of the coefficients closer to 1 indicate higher levels of reliability; Cooper & Schindler, 2008). The results in Table 1 indicate that the value of Cronbach's alpha for all of the latent variables was found above 0.7, which is above the required value of 0.7 (Chauhan, 2006).

**Table 1:** Reliability result

Factors	Cronbach's alpha (α)	Final number of indicators
Women entrepreneurship success	0.938	8
Business support	0.918	4
Access to finance	0.889	5
Saving practice	0.877	5
Skill development	0.854	4

### Data analysis

The data collected through the questionnaire were analyzed through the statistical package for social science (SPSS) 26 software. First, the data were analyzed with descriptive statistical analysis to provide frequency and percentage for respondents' interpretation and firm characteristics. Then the data were again put into factor analysis to examine the structural pattern among latent variables developed to measure microfinance services and women's entrepreneurship development using explanatory factor analysis (EFA). Further, the correlation analysis examined the relationship between the microfinance service and women's entrepreneurship development. Finally, regression analysis was used to find out the most dominant factor among the microfinance institution service offers that influences the development of women's entrepreneurship.

### Ethical considerations

This study does not include any human subjects experimentation, it is a pure survey, and the data were collected through a questionnaire. Thus, the only consent of respondents is required to participate. As a result, all participants have informed of the study's objective, and informed verbal consent was obtained prior to filling the questionnaire. In addition, privacy is ensured by using code instead of the subject name on the questionnaire to assure that the information given by each respondent is kept confidential. Further, the study was conducted according to the established ethical guidelines of Assosa University.

## Findings and results

### Response rate

In this study, the self-administered questionnaire was distributed to selected women Micro and Small enterprises operators of microfinance institutions in Assosa town. A total of 165 questionnaires were distributed, out of which only 125 were found valid and used for analysis giving a usable response rate of (76.2%). This response rate is considered sufficient, rational, and representative of the sample population.

### Respondents demographic profile

Along with many other constraints for the development, success, and long-term profitability of MSEs, the owner or manager's experience and education level have a significant impact. To know the owners' details, respondents were asked about their education level and years of experience in the business. The result in Table 2 indicates that 36.8% and 35.2% of respondents were aged "between" 20–34 and below 20 years, respectively. At the same time, the remaining 24% and 4% of respondents were aged "between" 35–50 and above 50 years, respectively. The above result indicates that the majority, i.e., 70% of the respondents, were below 35 years. The finding concludes that a significant representation of young and productive workforces is geared towards improving their living standards and the general development of their communities. When we examine the respondents' highest level of education, the findings show that half of the respondents, 50.4% and 26.4% have primary and secondary/high school level education, respectively.

**Table 2:** Respondents demographic profile

Variable	Description	Frequency	Percentage
Age	Between 20–34	46	36.8
	Below 20	44	35.2
	Between 35–50	30	24.0
	Above 50	5	4.0
Level of education	Elementary education	63	50.4
	Secondary/high school	33	26.4
	First degree and above	11	8.8
	Vocational school	10	8.0
	No formal education	8	6.4
Marital status	Married	85	68.0
	Single	28	22.4
	Divorced	12	9.6
Experience in business	Between 1–5 years	76	60.8
	Between 6–10 years	18	14.4
	Above 10 years	17	13.6
	Less than 1 year	14	11.2

In contrast, the remaining 8.8%, 8%, and 6.4% of respondents have a first degree, vocational education, and no formal education, respectively. The above finding means that most surveyed women have attained basic literacy skills and could understand and utilize the economic importance of microfinance services. The response of sampled women on their marital status and family size is shown in Table 1. The finding reveals that 68% of sampled women are married, 22.4% are divorced, and the remaining 9.6% of them were widowed, respectively. Finally, when we examine respondent's level of experience in the business, the result indicates that the majority, 60.8% of them are in the business between 1–5 years, 14.4% "between 6–10 years", 13.6% for more than 10 years, and 11.2% for not more than 1 year, respectively—Table 2 respondents' background profiles.

### Enterprise characteristics

Table 3 presents firm characteristics. The result indicates 48% of women-owned MSEs were established "between" 2011–2020, and 44% of them were joined the sector after 2020, respectively. The remaining 5.6% and 2.4% of enterprises were established "between 2000–2010" and before 2000. Concerning the type of businesswomen engaged, about 42.1% are engaged in the retail trade sector, and the service sector accounts for 25.6% of the sample enterprises, the manufacturing sector accounts for 14%, the construction sector accounts for 11.6% and 6.6% of surveyed women-owned businesses were other types of business. The enterprises' initial and current status results indicate that the majority, 107 (88.4%), were initially established at a micro-level. The remaining and only 14 (11.6%) of surveyed enterprises were established at a small-scale level. However, from the current status of these enterprises, it is noted that 83.5% were operating at the microscale level. This result indicates that, from the total of 107 enterprises initially established at the microscale level, only five enterprises were promoted to the next level, i.e., to the small-scale level. This is maybe due to a lack of continuous follow-up from local government agencies on the evaluation of growth and performance of MSEs to promote from one level to the other. Therefore, it shows the need for serious follow-up from local government to promote micro and small enterprises from micro to small, small to medium, and the like based on the growth of capital and employment level—Table 3 enterprises characteristics.

**Table 3:** Enterprises characteristics

Variable	Description	Frequency	Percentage
Enterprise year of establishment	Between 2011–2020	60	48.0
	After 2020	55	44.0
	Between 2000–2010 years	7	5.6
	Before 2000	3	2.4
Type of the business	Service	51	42.1
	Retail business	31	25.6
	Manufacturing	17	14.0
	Construction	14	11.6
	Others	8	6.6
Enterprise status during establishment	Micro	107	88.4
	Small	14	11.6
Enterprise current status	Micro	101	83.5
	Small	20	16.5

### Exploratory factor analysis (EFA)

To examine the suitability of the instrument developed to measure the variables for factor analysis, EFA using principal component extraction with Varimax rotation was performed for five variables, i.e., Women entrepreneurship success, business support, access to finance, saving practice, and Skill development to determine the structural pattern of the developed dimensions with the help of rotated component matrix, TVE, KMO, eigenvalues, and Bartlett's Test of Sphericity. From 29 items developed and subjected to explanatory factor analysis, only 26 of them were retained, and three items were eliminated because of cross-loadings. The three items discarded during EFA were AF6 (access to loans does not have too many conditions) from factor 2, BS4 (the savings withdrawal procedure is easy) from factor 3, and SD5 (the savings withdrawal procedure is easy) from factor 5, respectively. The 26 items retained after EFA yielded five factors explaining 70% of the cumulative variance. The results of the total variance explained are included in the Appendix section. Table 4 presents the factor loadings of the 26 items retained under the five extracted factors.

**Table 4:** Factor loadings

Rotated component matrix^a^						
Code	Item descriptions	Component				
		1	2	3	4	5
ED1	Profits of my enterprise tend to increase	0.761				
ED2	The number of workers in my business has begun to increase	0.842				
ED3	The number of products of my enterprise tends to increase	0.805				
ED4	The number of buyers of my venture tends to increase	0.835				
ED5	My household/family income tends to increase	0.778				
ED6	My household/family consumption tends to increase	0.745				
ED7	My household/family assets tend to increase	0.741				
ED8	My household/family savings tend to extend	0.792				
AF1	The loan interest rate is reasonable		0.759			
AF2	The loan obtaining procedure is simple		0.771			
AF3	The loan amount is sufficient		0.802			
AF4	The loan repayment period is sufficient		0.687			
AF5	The loan repayment procedure is easy		0.738			
BS1	The business support training helped me in proper business record keeping			0.732		
BS2	The business support training has improved and developed my saving culture			0.862		
BS3	The business support training improved my financial management knowledge			0.827		
BS5	The business support training given on marketing is sufficient and useful			0.732		
SV1	The savings interest rate is reasonable				0.732	
SV2	The saving procedures are simple and easy				0.663	
SV3	Savings enables access to other services such as loans from the MFIs				0.766	
SV4	The savings withdrawal procedure is easy				0.670	
SV5	The saving is mandatory				0.793	
SD1	My business benefits from skill development initiatives					0.764
SD2	The number of skill development programs is adequate					0.747
SD3	The Skill development programs are useful in improving my social status					0.769
SD4	The Skill development programs are useful in improving my family life					0.730

Extraction method: principal component analysis

Rotation method: varimax with Kaiser normalization

^a^Rotation converged in 5 iterations

### Mean analysis of the main variables

#### Access to finance

Table 5 summarizes the means and standard deviations of respondents' results related to women's access to microfinance services so far. Concerning the results obtained, the item "The loan repayment period is sufficient" was rated first with a mean of 3.56, and "Access to loans does not have too many conditions" was rated second with a mean value of 3.44 in a five-point Likert scale. This indicates the loan repayment period and conditions to obtain a loan were moderately sufficient. Whereas "The amount of the loan repayment period" scored a mean of 3.12 and "The loan obtaining procedure is simple" scored a mean value of 3.01, respectively. This indicated that the loan repayment and obtaining procedure are not simple for women. Finally, the item "The loan interest rate is reasonable" scored a mean value of 2.9, indicating the loan interest rate is not reasonable. Therefore, the average mean of the scale access to finance items was 3.18 on a five-point Likert scale. This low mean value generally indicates that access to finance or credit was low among surveyed women entrepreneurs. Specifically, the loan amount, procedures and conditions of repayment, repayment period, and loan interest rates were insufficient for women entrepreneurs. Table 5 details the summary of the above result.

**Table 5:** Access to finance (average mean = 3.18)

Items	*N*	Mean	Std. deviation
The loan repayment period is sufficient	125	3.56	1.066
Access to loans does not have too many conditions	125	3.44	1.210
The loan amount is sufficient	125	3.12	1.195
The loan repayment procedure is easy	125	3.10	1.149
The loan obtaining procedure is simple	125	3.01	1.195
The loan interest rate is reasonable	125	2.90	1.194

#### Business skill development program

Table 6 summarizes the means and standard deviations of respondents' results of MFI service related to business skill development service. The mean value of the item, "The skill development programs are useful in improving my social status", was 3.15, "The skill development programs are useful in improving my family life" had a mean score of 2.91, "The number of skill development programs is adequate" had a mean score of 2.89, and "My business benefits from skill development initiatives" had a mean score of 2.86, respectively. The result of the average mean score of the four items in the scale, i.e., the skill development training given by MFIs, is 2.95 on a five-point Likert scale. This low positive mean result shows that the skill development training given by MFI's was insufficient to help women entrepreneurs improve their personal and family life, respectively. Table 6 details the summary of the above result.

**Table 6:** MFI skill development initiative (average mean = 2.95)

Items	*N*	Mean	Std. deviation
The skill development programs help improve my social status	125	3.15	1.283
The skill development programs help improve my family life	125	2.91	1.218
The number of skill development programs is adequate	125	2.89	1.087
My business benefits from MFI's life skill development initiatives	125	2.86	1.180

#### Business support

Table 7 summarizes the means and standard deviations of respondents' results of MFI related to business support for women micro and small enterprise operators. The mean value of the item, "The business support training helped me in proper business record keeping" was 3.38, "The business support training given on marketing is sufficient and useful" had a mean score of 3.16, "The business support training improved my financial management knowledge" had a mean score of 3.09, and "The business support training has improved and develop my saving culture" had a mean score of 2.93, respectively. The result of the average mean score of the four items in the scale, i.e., the business support provided by MFIs, is 3.14 on a five-point Likert scale. This shows that the business support provided by MFI did not help women entrepreneurs to develop a saving culture, financial management skills, business record keeping, and did not help women entrepreneurs in proper networking for market access. Table 7 details the summary of the above result.

**Table 7:** MFI business support (average mean = 3.14)

Items	*N*	Mean	Std. deviation
The business support training helped me in proper business record keeping	125	3.38	1.024
The business support training given on marketing is sufficient and valuable	125	3.16	1.152
The business support training improved my financial management knowledge	125	3.09	1.132
The business support training has improved and developed my saving culture	125	2.93	1.137

#### Saving practice

Table 8 summarizes the means and standard deviations related to the role of MFI in improving women clients' saving practice. The mean value of the item, "The savings withdrawal procure is easy", was 3.38, "The saving is mandatory, and it improved my saving habit." had a mean score of 3.06, "The savings interest rate is reasonable" had a mean score of 2.98, "Savings enables access other services such as loans from the MFIs" had a mean score of 2.95, and "The saving procedures are simple and easy" had a mean score of 2.83 in a five-point Likert scale, respectively. The average mean score of the five items in the saving practice scale was 2.98 on a five-point Likert scale. This low average mean value indicates that the MFI's saving and withdrawal procedures are not simple. The saving interest rate was low, and the mandatory and compulsory/periodic savings did not significantly improve women's saving practice. Generally, the MFI service offered to women is unable to improve and change the saving practices of its clients. The institutions were also failed to achieve one of their primary goals, i.e., improving the livelihood of the poor, disadvantaged, and needy minority groups, including women, through improved saving practice. Table 8 details the summary of the above result.

**Table 8:** Women's saving practice (average mean = 2.98)

	*N*	Mean	Std. deviation
The savings withdrawal is procedure is easy	125	3.11	1.152
Saving is mandatory, and it improved my saving habit	125	3.06	1.109
The savings interest rate is reasonable	125	2.98	1.111
Savings enables access to other services such as loans from the MFIs	125	2.95	1.061
The saving procedures are simple and easy	125	2.83	1.155

#### Women's entrepreneurship development

Table 9 summarizes the means and standard deviations related to the role of MFI in improving women clients' entrepreneurship/micro and small enterprises development. The mean value of the item, "My family consumption tend to increase." was 3.66, "My family assets tend to increase" had a mean score of 3.58, "My household/family income tends to increase" had a mean score of 3.52, "Number of products of my enterprise tend to increase" had a mean score of 3.51, "Number of buyers of my venture tend to increase" had a mean score of 3.49, "Number of workers of my business begun to increase" had a mean score of 3.48, "Number of workers of my business begun to increase" had a mean score of 3.46, and "My household/family savings tend to extend" had a mean score of 3.35 in a five-point Likert scale, respectively. The average mean score of the eight items on the scale was 3.51 on a five-point Likert scale. This average mean value indicates that the overall MFI's services offered moderately improved women clients' family consumption, fixed assets size, income, and increased the type of products/services offered to customers, numbers of workers, profits, and the amount of saving, respectively. This result indicates that further improvement of the MFI service will play a significant role in developing women-owned micro and small enterprises and household income and consumption. Generally, more work should be done on the accessibility, amount, and rate of interest on the loan, provision of business-related training, and other skill development services to women clients for further success of their business and improved livelihood, respectively. Table 9 details the summary of the above result.

**Table 9:** Women entrepreneurship development (average mean = 3.51)

Items	*N*	Mean	Std. deviation
My household/family consumption tends to increase	125	3.66	1.033
My household/family assets tend to increase	125	3.58	1.072
My household/family income tends to increase	125	3.52	1.147
The number of products/services of my enterprise tends to increase	125	3.51	1.090
The number of buyers of my venture tends to increase	125	3.49	1.133
The number of workers in my business has begun to increase	125	3.48	1.126
Profits of my enterprise tend to increase	125	3.46	1.147
My household/family savings tend to extend	125	3.35	1.125

### Microfinance institution and women entrepreneurship development

A Pearson correlation analysis was employed to determine the relationship between microfinance institution service and women's micro and small enterprise development. The results of correlation coefficient between access to finance is positive (*r* = 0.463) with corresponding *P*-value (*P* = 0.00), skill and development (*r* = 0.315) with corresponding *P*-value (*P* = 0.00), and saving practice (*r* = 0.514) with corresponding *P*-value (*P* = 0.00) indicates the existence a significantly positive relationship between women entrepreneurial development, respectively, at *p* < 0.01 level of significance. Hence, hypotheses H_1_, H_2_, H_4_ were accepted. At the same time, the correlation coefficient between business support program and growth of women entrepreneurial development (*r* = 0.019) with corresponding *P*-value (*P* = 0.813) is greater than (*p* < 0.05) level of significance. Therefore, this very low positive correlation result indicates the absence of a statistically significant relationship between MFI's business support program and the growth of women's micro and small-scale business development among sampled Assosa town respondents. Therefore, H_3_ was rejected (Table 10).

**Table 10:** The correlation result

Correlations					
	AF	SD	BS	SV	WED
AF	1				
SD	0.334^**^	1			
BS	0.091	0.167^*^	1		
SV	0.383^**^	0.540^**^	0.250^**^	1	
WED	0.463^**^	0.315^**^	0.019	0.514^**^	1

*AF *access to finance, *SD *skill and development, *BS *business support, *SV *saving practice, *WED *women entrepreneurship development

*Correlation is significant at the 0.05 level (2-tailed)

**Correlation is significant at the 0.01 level (2-tailed)

Generally, the above result concludes that the MFI's services related to access to credit, skill development programs, and saving practice were positively related to the development and success of women client's business enterprises. The MFI's service has given them the chance to improve family consumption income, increase assets size products/services offered to customers, maximize profits, and effectively deal with the challenges facing while operating their business.

### The regression analysis

The multiple regression analysis was used to determine the dominant factors among the four independent variables (credit access, training, social capital, and saving services) and the dependent variable (growth of women entrepreneurs). When the predictor variables change independently, the standardized coefficient beta represents the coefficient of regression that analyzes a unit change in the predictor variables. The greater the β- value, the greater the impact of the independent variable on the dependent variable (Table 11).

**Table 11:** Multiple regression result

Coefficients^a^								
Model		Unstandardized coefficients		Standardized coefficients	*t*	Sig	Collinearity statistics	
		*B*	Std. error	Beta			Tolerance	VIF
1	(Constant)	1.258	0.367		3.429	0.001		
	Access to finance	0.302	0.072	0.300	4.181	0.000	0.791	1.265
	Skill and development	0.002	0.077	0.002	0.021	0.983	0.685	1.459
	Business support	− 0.071	0.084	− 0.058	− 0.855	0.394	0.891	1.122
	Saving service	0.406	0.079	0.413	5.111	0.000	0.623	1.606

^a^Dependent variable: women entrepreneurship development

By examining the β-value for all four independent variables, it was determined that the MFI saving service with (*β* = 0.413, *P* = 000) has the strongest influence on women's entrepreneurship development. This means that when the variance explained by three variables in the model is controlled, the MFI's saving service makes the strongest unique contribution to women's entrepreneurship development. Further, access to credit was the second most determinant factor that affects women's entrepreneurship development with (*β* = 0.3, *P* = 000). However, the standardized coefficient beta result of (*β* = 0.002, *P* = 983) and (*β* = − 0.071, *P* = 0.394) revealed the skill and development and business support, i.e., the MFI's non-financial service offer has no significant influence on women's entrepreneurship development. Further, the adjusted *R* square of 0.350, Table 12 shows that the proportion of the variations in women's entrepreneurship development is explained by the four independent variables (saving practice, business support, access to finance, skill and development) jointly is 33.4%. The remaining 66.6% of the variance is explained by other variables not included in this study.

**Table 12:** Regression model summary

Model summary^b^									
Model	*R*	*R* square	Adjusted *R* square	Std. error of the estimate	Change statistics				
					*R* square change	*F* change	*df*1	*df*2	Sig. *F* change
1	0.592^a^	0.350	0.334	0.86393	0.350	21.573	4	160	0.000

^a^Predictors: (constant), saving service, business support, access to finance, skill and development

^b^Dependent variable: women entrepreneurship development

Table 13 shows the ANOVA test on the general significance of the model. The result revealed that saving practice, business support, access to finance, skill, and development were found statistically significant at predicting women entrepreneurship development. *F* statistic = 21.573 and *P*-value (Sig. = 0.000) is less than the *p *value of 0.05. Therefore, H_5_ was accepted. This result indicates that microfinance institutions' financial and non-financial services are highly significant and the hypothesized determinants of women's entrepreneurship development.

**Table 13:** The ANOVA output

ANOVA^a^						
Model		Sum of squares	*df*	Mean square	*F*	Sig
1	Regression	64.405	4	16.101	21.573	0.000^b^
	Residual	119.419	160	0.746		
	Total	183.823	164			

^a^Dependent variable: women entrepreneurship development

^b^Predictors: (constant), saving practice, business support, access to finance, skill and development

## Conclusions

The descriptive mean result shows that access to finance or credit was low and challenging among surveyed women entrepreneurs. Specifically, the loan amount was insufficient, procedures and conditions of repayment were not easy, the repayment period was not adequate, and loan interest rates were very high for women entrepreneurs. The result concerning the saving service was not satisfactory, and the withdrawal procedures are not easy for women clients of the enterprises. According to surveyed women entrepreneurs, the procedures of saving and withdrawing money are not simple, the saving interest rate was low, and the mandatory and compulsory/periodic savings did not significantly improve women's saving practice. Generally, the MFI service offered to women is unable to improve and change the saving practices of its clients. The institutions also failed to achieve one of their major goals, i.e., improving the livelihood of the poor, disadvantaged, and needy minority groups, including women through improved saving practice.

Furthermore, the skill development training service provided by MFIs was insufficient to assist women entrepreneurs in improving their personal and family lives. This finding indicates that MFIS training did not help women entrepreneurs develop a saving culture, improve product quality financial management skills, or keep proper records. Finally, the business support provided by MFI did not help women entrepreneurs to improve their social status, lifestyle, access to market, communication skills, and they are not adequate at all. The correlation analysis findings indicated a positive and significant association between saving practice, access to credit, skill development training, and the development of women entrepreneurs. However, the business support program was not significantly related to the development of women entrepreneurs among sampled respondents in Assosa town. However, when we examine the result of the regression analysis, saving and the credit or loan services of the MFI have the strongest influence and make the strongest unique contribution to women's entrepreneurship development. At the same time, skill and development and business support have no significant influence on women's entrepreneurship development. This indicates the financial services offered of the MFI are making the strongest contribution to the development of women entrepreneurs as that of non-financial services.

## Implications of the study

Microfinance is emerging as a potential tool for poverty alleviation in developing economies. As a result, the majority of microfinance programs have a clear goal of reducing poverty and empowering women. However, despite increasing the number of microfinance institutions, access to finance is still regarded as one of the most significant barriers to female entrepreneurs. According to research, investing in women is the most effective way to improve health, nutrition, hygiene, and educational standards for families and thus for society as a whole. However, the operation of rural women's entrepreneurship entails significant risks, hard work, enormous sacrifice, and sincerity of purpose, all of which overcome numerous obstacles. Therefore, we should temper our expectations of the impact of promoting women's entrepreneurship as a new engine of growth, bringing gender equality, social change, and economic development. As a result, special assistance for women in financial and non-financial services is required. Therefore, formal government policy to create an appropriate environment that supports female entrepreneurs with resources to assess business feasibility and legal counsel are required at each stage of starting a new business.

## Data Availability

The data relating to this study will be readily available upon request for any validation purpose.

## Appendix

### KMO and Bartlett's test

**Table:**

KMO and Bartlett's test		
Kaiser–Meyer–Olkin measure of sampling adequacy		0.801
Bartlett's test of sphericity	Approx. Chi-square	2802.184
	*df*	325
	Sig.	0.000

### Total variance explained

**Table:**

Total variance explained									
Component	Initial Eigenvalues			Extraction sums of squared loadings			Rotation sums of squared loadings		
	Total	% of variance	Cumulative %	Total	% of variance	Cumulative %	Total	% of variance	Cumulative %
1	5.322	20.469	20.469	5.322	20.469	20.469	4.944	19.015	19.015
2	4.062	15.622	36.091	4.062	15.622	36.091	3.637	13.990	33.006
3	3.900	15.001	51.092	3.900	15.001	51.092	3.537	13.604	46.609
4	2.682	10.313	61.405	2.682	10.313	61.405	3.106	11.945	58.555
5	2.236	8.598	70.004	2.236	8.598	70.004	2.977	11.449	70.004
6	0.982	3.779	73.782						
7	0.828	3.185	76.968						
8	0.702	2.702	79.669						
9	0.562	2.160	81.829						
10	0.492	1.891	83.720						
11	0.449	1.729	85.449						
12	0.422	1.625	87.074						
13	0.371	1.427	88.501						
14	0.355	1.364	89.865						
15	0.333	1.280	91.144						
16	0.314	1.209	92.353						
17	0.290	1.114	93.468						
18	0.265	1.021	94.489						
19	0.251	0.965	95.454						
20	0.224	0.862	96.316						
21	0.204	0.786	97.103						
22	0.181	0.698	97.800						
23	0.174	0.671	98.471						
24	0.151	0.579	99.050						
25	0.135	0.518	99.568						
26	0.112	0.432	100.000						

Extraction method: principal component analysis

## Abbreviations

AF: Access to finance
BS: Business support
EFA: Explanatory factor analysis
KMO: Kaiser–Meyer–Olkin
MFI: Microfinance institutions
MSE: Micro and small enterprises
NGO: Non-governmental organizations
SD: Skill and development
SPSS: Statistical Package for Social Science
SV: Saving practice
TVE: Total variance explained
UK: United Kingdom
WED: Women entrepreneurship development
